# A cross-sectional study of reproductive performance of improved dairy cattle in smallholder dairy farming in Tanzania

**DOI:** 10.1007/s11250-026-05301-5

**Published:** 2026-09-09

**Authors:** Athanas Ngou, Richard Laven, Timothy Parkinson, Isaac Kashoma, Daniel Donaghy

**Affiliations:** 1 School of Veterinary Science, Massey University, Private Bag 11 222, Palmerston North, 4442 New Zealand; 2 College of Veterinary Medicine and Biomedical Sciences, Sokoine University of Agriculture, Morogoro, 67 115 Tanzania; 3 School of Agriculture and Environment, Massey University, Private Bag 11222, Palmerston North, 4442 New Zealand

**Keywords:** Smallholder dairy farming, Reproductive performance, Farm records keeping, Pregnancy diagnosis, Tanzania

## Abstract

A generalised lack of reliable records from smallholder dairy cattle farms in Tanzania is a significant impediment to the assessment of herds reproductive performance. A large-scale study of smallholder dairy herds was undertaken to identify the methods of recording that were in use, their effectiveness, and whether there are regional variations in herds’ reproductive performance. Records were examined from 1279 dairy cows from 322 farms across the 6 main dairy-farming regions of Tanzania. For calving to first service (CFSI: *n* = 49 (47%)), calving to conception (CCI: *n* = 313 (24%)) and inter-calving (ICI: *n* = 424 (33%)) intervals. Data were analysed with respect to region. Mean (± IQR) intervals were: CFSI: 249 (87–303), CCI: 268 (111–334) and ICI: 563 (398–686) days, respectively. All breeding dairy cattle (*n* = 1279) on these farms were examined for pregnancy status by transrectal ultrasound. Pregnancy was detected in 586/1279 (46%) cattle, with the highest and lowest pregnancy rates occurring in the Mbeya (55%) and Tanga (37%) regions, respectively. Reproductive performance of smallholder dairy cattle was generally poor, being characterised by low pregnancy rates, and long CFSI, CCI and ICI. Record-keeping practices were generally unsatisfactory, largely relying on farmer recall; with recorded data available for only ~ 50% of cattle. These findings highlight the urgent need for improved record-keeping systems and reproductive management at the farm level. Expanding access to accurate pregnancy diagnosis, to provide a “proportion pregnant” might be useful as an interim reproductive performance indicator until record-keeping improves. Strengthening record-keeping and could significantly improve reproductive management and decision-making.

## Introduction

Nearly all milk produced in Tanzania originates from smallholder dairy farms, which typically operate under low-technology, low-input and low-output systems (Brett 2019). As a result, despite Tanzania possessing the second-largest national cattle herd in Africa (World Bank, 2024), the country still faces a substantial milk deficit of nearly 9 billion litres (Kolumbia 2024; MLF, 2024). This shortfall highlights the pressing need to boost the productivity of Tanzanian cattle, particularly those managed by smallholder farmers. One key constraint on smallholder dairy farms is poor reproductive performance, which markedly reduces milk production, longevity and lifetime productivity, while increasing involuntary culling and production costs (Shalloo et al. 2014). Furthermore, it results in significant non-monetary costs, such as reduced food security within the community, increased poverty, and reduced social status (Banda and Tanganyika 2021). The loss of these non-monetary benefits is particularly crucial in regions such as sub-Saharan Africa, where the social value of livestock is often greater than its monetary value (Chisoni et al. 2025). Improving reproductive performance on Tanzanian dairy farms is thus likely to have significant benefits for smallholder dairy farmers and their communities.

In order to develop a program for improving reproductive performance, there needs to be a baseline against which the impact of any changes can be evaluated. However, up-to-date information on the reproductive performance of smallholder dairy cattle in Tanzania is scarce (Allan et al. 2024), and even the older published sources were limited in their geographical extent (Mdoe 1993; Kanuya et al. 2000; Msanga et al. 2000; Lyimo et al. 2004; Swai et al. 2005b; Allan et al. 2024). Thus, up-to-date information is needed on the reproductive performance of smallholder dairy herds throughout Tanzania to provide a usable baseline for future improvements. Such information will also help to identify any differences in reproductive performance across regions, thereby enabling assessment of whether region-specific targets and programs are necessary. Obtaining these base-line data is, in turn, predicated upon identifying an effective method for measuring reproductive performance for Tanzanian smallholder dairy farms.

The present study was therefore designed to measure the reproductive performance of dairy cattle in smallholder dairy farming systems across Tanzania. It focused on (i) the evaluation of available farm records, and (ii) the reproductive examination of individual animals to establish their pregnancy status.

## Methodology

This survey was conducted simultaneously with the study described by Ngou et al. (2025). A brief outline of the methodology is included here.

### Ethical considerations and approval

The research was approved by the Ministry of Livestock and Fisheries through the Ethics Review Board of the Tanzania Livestock Research Institute (TALIRI) (reference number TLRI/RCC.21/007). Informed written consent was obtained before data collection, and the results were anonymised.

### Study area, selection of study farms and animals

**Fig. 1:**
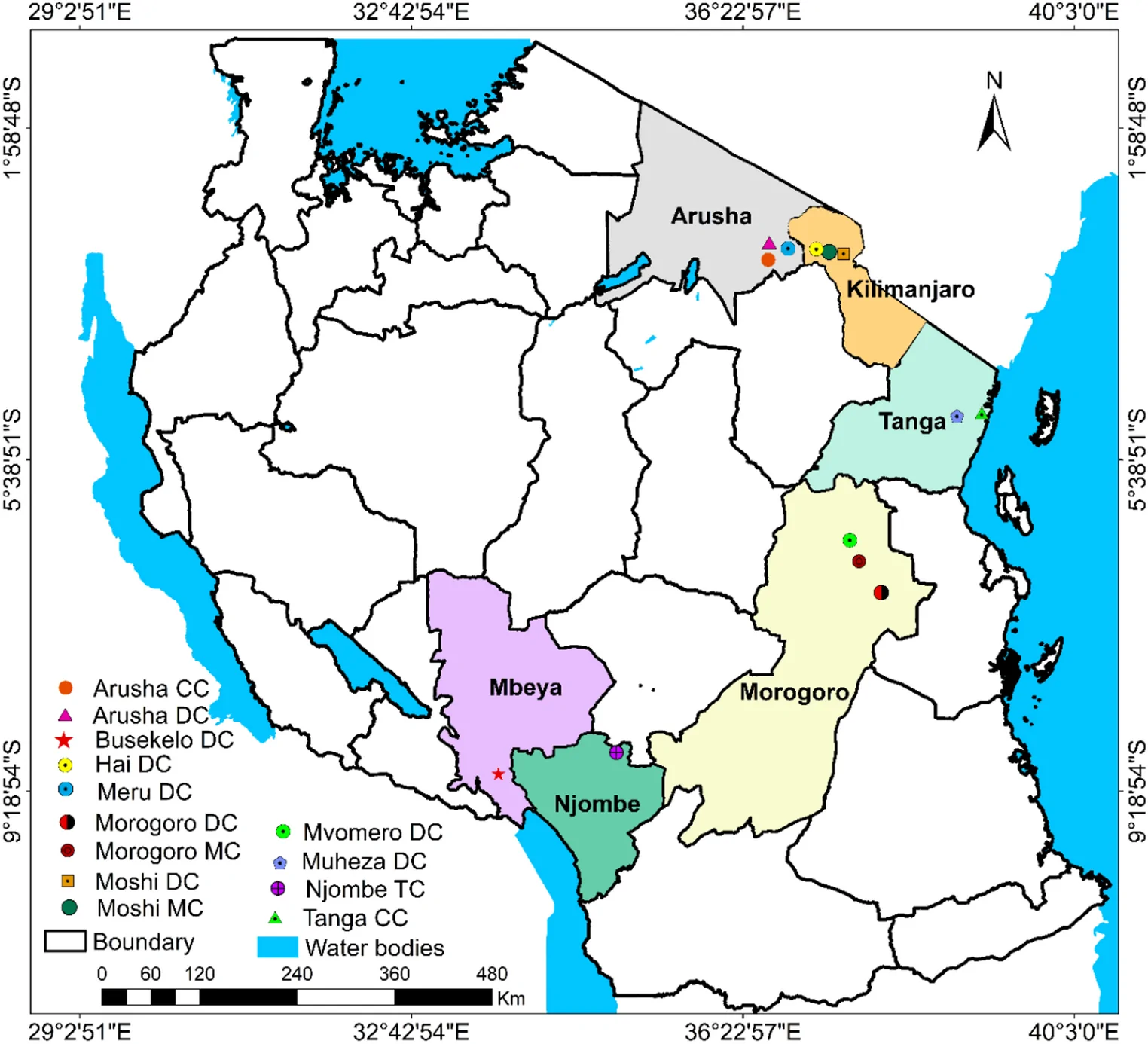
Map of Tanzania showing study regions and districts: Southern Highland regions, Northern Highland regions and Morogoro. NB: CC - City council, DC - District council and TC - Town council

A cross-sectional study was conducted between May 2022 and February 2023 in the six major dairy producing regions of Tanzania (Arusha, Kilimanjaro, Mbeya, Morogoro, Njombe and Tanga) (See Fig. 1). These regions were chosen due to their high proportions of improved dairy cattle (NBS&OCGS, 2021). Within each region, a convenience sampling technique was used to identify study districts, villages and the first farm in each village. Thereafter, a snowball sampling technique was used to select further study farms in that village. All of the farmers who participated in the present study had also participated in the survey of reproductive knowledge reported by Ngou et al. (2025).

### Field data collection

All data were collected and animal examinations were performed by the first Author (A.N.), with the assistance of the local veterinarian and/or livestock officer. Upon arrival at the farm, the veterinarian or livestock officer introduced the researcher to the farmer/respondent (usually the family head). The farmer was then asked to provide all reproduction-related records for the individual animals on the farm. Data collection was conducted by the Author, and was verified by the local veterinarian or livestock officer.

Data for animal identity, breeding and calving date(s), and parity were collected for each individual mature female animal (i.e., all cows that had calved at least once and all heifers considered by the respondent to be mature enough for breeding). Where date-specific information was absent, the farmer was asked directly about the missing information, using associated events such as religious observances (e.g., Eid and Christmas), school or public holidays, or the presence of any visitors on the farm, as an aide-memoire of when an event had occurred.

Body condition score (BCS; 1 to 5 scale: Wildman et al. (1982), body weight (heart girth measurements using a Rondo measuring tape) and pregnancy status were also assessed. Pregnancy status was determined using transrectal ultrasonography (Veterinary Ultrasound Scanner - CD66V, Wuhan Darppon Medical Technology, China). All records, whether recorded or provided by the farmer, were cross-checked against the outcomes of the pregnancy examination of the individual cows. For example, if the service date did not match the estimated gestational age, the farmer was further questioned in an effort to obtain more accurate information. Many farmers asked for the estimated gestational age to remind them of the service date or to predict the expected calving month.

### Definition of reproductive parameters

Indices of reproductive performance were based upon established definitions (Noakes 1997; Hudson et al. 2018), with slight modifications to suit the focus of the present study. They were calculated as follows:

i. Inter-calving interval (ICI): interval between successive calvings;
ii. Calving to first service interval (CFSI): number of days from the most recent calving to the date of the first recorded (or remembered) insemination of the cow after that calving;
iii. Calving-to-conception interval (CCI): number of days from the most recent calving to the service that had resulted in the pregnancy detected by transrectal ultrasonography. This was usually the last recorded (or remembered) service (artificial insemination (AI) or bull), but this could be reviewed if the dates did not match the gestational age;
iv. First service to conception interval (FSCI): number of days between the first recorded (or remembered) service and the service that was identified as resulting in pregnancy;
v. Pregnancy status (pregnant or non-pregnant) at the time of the farm assessment. Cows that were not detected as pregnant at the visit, but which had been inseminated within the past 28 days, were re-examined, if possible, when 28 days had passed. If re-examination was not possible, the cow was recorded as not pregnant;
vi. The voluntary waiting period (VWP): number of days allowed by the farmer between a cow calving and attempting to breed her again.

### Data management

Data were entered into an Excel spreadsheet (Microsoft, Seattle, USA). Descriptive statistics (mean, median, range and 95% confidence interval (CI)) were calculated for each index for the entire population and for each region. The effects of region on VWP, ICI, pregnancy status and CCI were then analysed. For the VWP, a generalised linear model, with VWP as an ordinal outcome and region as the predictor variable was used. For ICI, a generalised estimating equation model was used, with log_10_ ICI as the linear outcome variable, farm as the subject variable, cow identification (cowID) as the within-subject variable, and region as the predictor variable. For pregnancy status, a generalised estimating equation model was used, with pregnancy status (yes/no) as the binomial outcome variable, farm as the subject variable, cowID as the within-subject variable and region as the predictor variable.

For CCI, data were analysed using a mixed-effects Cox proportional hazard model, with farm as the random effect and region as a fixed effect. The output of this model was a hazard ratio with Tanga as the reference category.

The data for the 516 cows that were non-pregnant but had a known previous calving date were censored at the interval between that calving and the date of the pregnancy examination (i.e. as time to conception was unknown). The association between VWP and CCI was analysed using a linear model with the log_e_ average CCI for individual farms as the outcome variable and the reported VWP for the farms as the predictor variable.

For inter-regional comparisons, differences were considered of significance where the 95% CI of the hazard ratio (HR) with respect to the Tanga region did not include 1. Thus, for example, the hazard ratios for ICI are presented as the ratio of the hazard of conceiving for cows in the non-reference region with respect to the Tanga region, such that the hazard of conceiving represents the immediate chance (risk) of a cow conceiving at a given timepoint. Other hazard rations are reported similarly.

All analyses were undertaken using SPSS version 29 (IBM, New York, USA), except for the mixed-effect Cox regression, which was analysed using R (RStudio, Boston, USA).

## Results

Data were gathered for a total of 1279 cows from 322 farms across the six primary dairy-farming regions of Tanzania. Most farmers maintained their farm and animal records using basic and easily accessible methods, for example, noting them down on paper or, in some cases, writing directly on walls. Whilst these practices were common, inconsistencies and missing records were frequently encountered which limited the ease and completeness of data retrieval during the data collection. For example, over 50% of animals had only one recorded service date (typically the last one that resulted in pregnancy), with earlier services apparently being undocumented.

### Basic herd data

Numbers of farms and animals enrolled per region are given in Table 1. The average number of cows and heifers per farm across all regions was 4.0, with 2.6 animals per farm in milk. Tanga and Morogoro had higher numbers in each category than did other regions.

**Table 1:** Descriptive date of numbers of herds and cows by region

Region	Farms (% of entire survey)	Total cows (% of all animals in survey)	Total heifers# (% of all animals in region #)	Dairy cows and heifers# per farm	Total cows in milk (% of all animals in region)	Cows in milk per farm
Arusha	16 (5)	43 (3)	14 (33)	2.7	26 (60)	1.6
Kilimanjaro	65 (20)	219 (17)	48 (22)	3.7	160 (73)	2.5
Mbeya	69 (21)	217 (17)	63 (29)	3.1	119 (55)	1.7
Morogoro	60 (19)	279 (22)	41 (15)	4.7	200 (72)	3.3
Njombe	56 (17)	139 (11)	28 (20)	2.5	92 (66)	1.6
Tanga	56 (17)	382 (30)	64 (17)	6.8	245 (64)	4.4
Total	**322 (100)**	**1279 (100)**	**258 (20)**	**4.0**	**841 (66)**	**2.6**

# Nulliparous cattle

Bodyweight, condition score, parity and milk yield of cows are summarised by region in Table 2. Overall mean and median body weights were 346 and 344 kg (ranging from 150 to 650 kg), respectively. The overall mean and median BCS were both 3.0 (range 1.5 to 4.5). There were negligible differences between regions.

**Table 2:** Bodyweight, condition score, parity and milk yield by region

Region	Total cows per region	Bodyweight (kg)*		Body Condition Score (1–5)*		Parity			Milk yield per cow (L/cow/day)		
		Mean	Median (range)	Mean	Median (range)	Records (%#)	Mean	Median (range)	Records (%#)	Mean	Median (range)
Arusha	43	340	350 (180–500)	3.0	3.0 (2.0.5-4)	29 (100)	3.0	2 (1–7)	26 (93)	14.6	12 (8–30)
Kilimanjaro	219	356	350 (150–510)	3.0	3.0 (2.0–4.0)	169 (99)	2.6	2 (1–10)	160 (95)	13.1	12 (2–30)
Mbeya	217	344	337 (192–562)	3.0	3.0 (1.5-4.0)	106 (78)	2.8	2 (1–12)	119 (84)	13.5	14 (2–28)
Morogoro	279	335	350 (175–475)	3.0	3.0 (1.5-4)0.0	202 (87)	2.3	2 (1–7)	200 (86)	12.1	12 (4–35)
Njombe	139	335	333 (192–506)	2.9	3.0 (1.5–4.5)	106 (96)	2.6	2 (1–11)	92 (86)	14.7	15 (6–24)
Tanga	382	354	344 (192–650)	3.1	3.0 (1.5-4.0)	316 (100)	2.8	2 (1–10)	244 (81)	11.3	10 (3–34)
Total	**1279**	**346**	**344 (150–650)**	**3.0**	**3.0 (1.5–4.5)**	**928 (93)**	**2.7**	**2 (1–12)**	**841 (86)**	**12.6**	**12 (2–35)**

* All cows included in these data # %of cows in region

Overall median parity (for lactating cows and cows that were known to have been lactating) was 2 (ranging from 1 to 12). Parity records were not available for 7% of cows, primarily due to cows being purchased without prior reproductive history. The other main reason for missing parity data was poor record-keeping. The percentage of adult cows with no parity records varied across regions, ranging from 0% (Tanga and Arusha) to 22% (Mbeya). Overall, 20% of the cattle that were pregnancy tested were nulliparous (heifers). The proportion of heifers varied by region, ranging from 15% (Morogoro) to 33% (Arusha).

Milk yield records were available for 841 cows. No information on milk yield was available for 14% of eligible cows (excluding nulliparous heifers). Overall, the mean and median reported milk yields were 12.6 and 12 L/day, respectively (range: 2 to 35 L/day). There were small differences in median reported milk yield across regions, which varied from 11 L/cow/day (Tanga) to 15 L/cow/day (Arusha and Njombe). Regional differences were not statistically significant.

### Reproductive performance

Reproductive performance data are summarised in Tables 3, 4, 5 and 6. The proportions of cows with data available to calculate individual parameters ranged from 100% for pregnancy status (as all eligible cows were pregnancy-tested) to 25% for CCI.

**Table 3:** Voluntary waiting period across farms in different regions

Region	Farms (*n*)#	Mean	Median (IQR)	Range	OR (95% CI)†
Arusha	16	127.5	105 (79–161)	75–255	4.18 (1.77–13.1)
Kilimanjaro	65	113.0	90 (75–120)	60–360	1.99 (1.05–3.76)
Mbeya	69	72.5	60 (45–90)	35–180	0.22 (0.11–0.43)
Morogoro	60	119.7	105 (75–135)	45–303	3.88 (1.97–7.63)
Njombe	56	96.2	90 (90–90)	90–150	2.03 (1.11–3.73)
Tanga	56	92.5	90 (75–90)	45–195	Reference
Overall	**322**	**99.6**	**90 (75–105)**	**35–360**	

# VWP is reported on a per farm basis

Data for herd VWP are given in Table 3. A total of 178 farms reported a VWP and had at least one cow with a CCI (known last calving date and pregnant). Median herd VWP was 90 days (range: 35 to 360 days). Compared to the Tanga region, only farms in the Mbeya region had lower odds (Odds Ratio (OR): 0.22, 95% CI: 0.11–0.43) of having a longer herd VWP. Farms in the other regions had higher odds of having a longer VWP.

Calving to first service interval (CFSI) data (Table 4) were available for 595 cattle. Overall median CFSI was 166 days (range: 28 to 1822 days), with 25% of cows having CFSI of 303 days or more. The median and the upper quartile data for CFSI are much longer than the median VWP, indicating that farmers were reporting the most recent insemination rather than the first one.

**Table 4:** Data for key reproductive parameters of cows by region

Region	Number of recorded animals per region (%)#	Mean	Median (interquartile)	Range	Hazard ratio (95% confidence interval)
**Calving to First Service Interval (CFSI)**					
Arusha	22 (4)	348.9	136 (136–186)	80-1548	1.01 (0.64–1.60)
Kilimanjaro	107 (18)	336.4	210 (132–484)	33-1621	1.47 (0.91–2.37)
Mbeya	76 (13)	232.3	142 (72–297)	41-1151	1.41 (0.89–2.23)
Morogoro	114 (19)	240.7	167 (87–298)	28-1377	1.59 (0.98–2.57)
Njombe	73 (12)	215.8	131 (93–243)	35-1320	1.60 (1.03–2.50)
Tanga	203 (34)	215.0	144 (62–281)	29-1822	Reference
**Overall**	595 (46.5)*	**249.0**	**166 (87–303)**	**28-1822**	
**Calving to Conception Interval (CCI)**					
Arusha	10 (3)	233.4	189 (174–276)	97-1548	1.24 (0.43–3.54)
Kilimanjaro	58 (19)	264.5	254 (148–479)	54-1105	0.55 (0.28–1.07)
Mbeya	44 (14)	177.1	184 (80–408)	41-1151	1.61 (0.85–3.05)
Morogoro	75 (24)	187.6	176 (104–301)	46-1377	1.32 (0.72–2.42)
Njombe	39 (12)	190.2	173 (104–318)	59-1361	2.86 (1.4–5.85)
Tanga	87 (28)	179.7	175 (111–297)	38–880	Reference
**Overall**	313 (24.5)*	**268.3**	**189 (111–334)**	**38-1548**	
**Inter Calving Interval (ICI)**					
Arusha	17 (4)	509.6	445 (405–614)	350–1253	1.03 (0.69–1.55)
Kilimanjaro	72 (17)	633.2	638 (456–801)	283–1531	0.62 (0.50–0.78)
Mbeya	67 (16)	482.7	430 (369–568)	286–1425	1.14 (0.91–1.44)
Morogoro	78 (18)	582.2	559 (425–776)	301–1389	0.75 (0.60–0.93)
Njombe	39 (9)	457.7	406 (382–512)	350–949	1.59 (1.20–2.12)
Tanga	151 (36)	489	447 (385–567)	2,881,265	Reference
**Overall**	424 (33.1)*	**563.4**	**468 (398–686)**	**283–1531**	

# of animals in region * proportion of entire study cohort for which data were available

Difference in the estimated marginal means (95% CI) between regions are back transformed from the log_10_ estimated marginal means)

Similarly, first service to conception interval (FSCI) was evaluated in 492 animals (Table 5). The stated FSCI ranged from 0 to 477 days, with 359/492 (73%) of cows having a FSCI of 0 days. This means that, among the cows for which FSCI was reported, 73% would have become pregnant to that first service. This is extremely unlikely, especially in tropical conditions, as monitored first service conception rates generally vary between 25 and 50% (Kanuya et al. 2014; Tadesse et al. 2022). This infers those farmers were remembering the most recent service rather than the first service. No further analysis of first service data was therefore undertaken.

**Table 5:** First service to conception interval of cows by region

Region	Number of recorded animals per region (%)#	Mean	Median (IQR)	Range	Number with zero FSCI (% of cows in region)
Arusha	14 (33)	20.4	0 (0–2)	0-209	11 (79)
Kilimanjaro	93 (42)	24.6	0 (0–15)	0-477	69 (74)
Mbeya	110 (51)	15	0	0-333	93 (85)
Morogoro	119 (21)	20.6	0 (0–22)	0-229	86 (72)
Njombe	59 (42)	18.3	0	0-181	45 (76)
Tanga	97 (25)	20.4	0 (0–35)	0-148	55 (57)
Overall	492 (38)*	**20**	**0 (0–21)**	**0-477**	**359 (73)**

# %of cows in region * proportion of entire study cohort for which data were available

Calving to conception interval (CCI) data (Table 4) were available for 313 cattle (i.e. 313 cattle were pregnant and had a known previous calving date). The overall mean and median values were 268 and 189 days respectively (range: 38 to 1548 days). Between regions, only animals from the Njombe region had a clearly higher hazard of pregnancy (2.86, 95% CI: 1.4–5.85) than cows from Tanga. Other regions did not differ significantly compared to Tanga.

Inter-calving interval (ICI) data were available for 424 cows (Table 4). Across all regions, the mean and median ICI were 563 and 468 days, respectively (range: 283–1531 days). Cows in Kilimanjaro and Morogoro had lower hazard of calving, with hazard ratios if 0.62 (95% CI: 0.50–0.78) and 0.75 (95% CI: 0.60–0.93), respectively, indicating longer ICI intervals. Conversely, cows in Njombe had a higher hazard of calving than those in Tanga (HR = 1.59, 95% CI: 1.20–2.12), indicating shorter ICI. For the remaining regions, ICI data did not differ significantly compared to Tanga.

Comparison of the VWP and average CCI of individual farms showed that a longer VWP was associated with an increased average CCI, with a 10-day increase in VWP increasing herd-average CCI by 1.02 (95% CI 1.01 to 1.04) times. This model predicted that, for a farm with a VWP of 50 days, expected average CCI would be 187 days, whilst for a farm with a VWP of 125 days, the expected average CCI would be 217 days (90 days longer). However, the model also identified that the VWP for individual farms accounted for only 4% of the variance in their CCI.

Pregnancy status was established for 1279 cattle. Of these, 588 (46%) were pregnant (443 (76%) cows, 143 (24%) heifers: Table 6). The pregnancy rate in nulliparous heifers was 143/258 (55%), versus 443/1021 in (43%) parous cows. Thus, across all regions, the relative risk of a heifer being pregnant was 1.3 (95% CI: 1.1 to 1.5) times higher than that of a parous cow. Across the regions, the odds of being pregnant were higher in Mbeya (OR: 2.07, 95% CI: 1.47–2.9), Morogoro (OR: 1.81, 95% CI: 1.32–2.47) and Njombe (OR: 1.56, 95% CI: 1.05–2.3) than in Tanga. There were too few cows examined in Arusha to accurately determine the likely difference in odds of being pregnant compared to Tanga. Much of the difference in pregnancy status between regions seemed to have depended upon the reproductive performance of heifers. In adult cows alone, only Morogoro had a clear difference in odds of pregnancy compared to Tanga (OR 1.71; 95% CI 1.22–2.4), whereas for heifers, all regions (except for Arusha) had an OR whose 95% CI did not include 1 (Table 6). For all regions except Morogoro and Tanga, the raw proportion of pregnant animals was higher in heifers than in cows, and for all regions except Morogoro, the difference in proportion of cows and heifers that were pregnant was ≥ 9%. The largest difference was seen in Mbeya, where the proportion of pregnant heifers was 34% (95% CI 25.4–49.5%) higher than the proportion of pregnant cows. For adult cows only, the odds of a cow being pregnant were higher (OR: 1.71, 1.22–2.4) in Morogoro than in the Tanga region. For heifers only, the odds of being pregnant were higher in Mbeya, Kilimanjaro, Njombe and Morogoro with OR (95% CI) of 12.1 (5.17–28.2), 4.26 (1.92–9.47), 3.95 (1.55-10) and 2.43 (1.07–5.52), respectively, compared to the Tanga region.

**Table 6:** Comparison between the proportion of pregnant dairy cattle, cows, and primiparous heifers by region

Region	Total number of dairy cattle in survey	Pregnant (% of all dairy cattle in the region)	OR (95% Confidence Interval)	Total cows (% of all dairy cows in region)	Pregnant cows (% of all dairy cows in region)	OR (95% Confidence Interval)	Total heifers* (% of all cattle in region)	Pregnant heifers (% of all heifers in region)	OR (95% Confidence Interval)
Arusha	43	16 (37)	0.99 (0.52–1.9)	29 (67)	10 (34)	0.81 (0.37–1.81)	14 (33)	6 (43)	1.92 (0.58–6.3)
Kilimanjaro	219	95 (43)	1.28 (0.91–1.8)	171 (78)	65 (38)	0.95 (0.65–1.39)	48 (22)	30 (63)	4.26 (1.92–9.47)
Mbeya	217	120 (55)	2.07 (1.47–2.9)	154 (71)	68 (44)	1.22 (0.83–1.8)	63 (29)	52 (83)	12.1 (5.17–28.2)
Morogoro	279	145 (52)	1.81 (1.32–2.47)	238 (85)	125 (53)	1.71 (1.22–2.4)	41 (15)	20 (49)	2.43 (1.07–5.52)
Njombe	139	67 (48)	1.56 (1.05–2.3)	111 (80)	50 (45)	1.27 (0.82–1.96)	28 (20)	17 (68)	3.95 (1.55-10)
Tanga	382	143 (37)	Reference	318 (83)	125 (39)	Reference	64 (17)	18 (28)	Reference
Overall	**1279 (100)**	**586 (46)**		**1021 (80)**	**443 (43)**		**258 (20)**	**143 (55)**	

Key: OR: odds ratios derived from binary logistic regression. * nulliparous animals

## Discussion

Smallholder dairy farming plays a crucial role in enhancing livelihoods, improving human nutrition and promoting rural development in developing countries such as Tanzania (McDermott et al. 2010). Smallholder dairy farming in Tanzania remains largely underdeveloped and is characterised by low-input/low-tech and low-output systems (Hemme and Otte 2010; Maleko et al. 2018a) that result in low productivity. The present study, the first to be undertaken on a pan-national basis, aimed to assess the productivity and reproductive performance of smallholder dairy cattle farms across the six main dairy-producing regions of Tanzania, as part of an overall evaluation of smallholder dairy cattle farming in the country. Constraints on dairy cows’ yield and reproductive performance, as perceived by their smallholder farmer owners, were described in Ngou et al. (2025), so the present study concentrated on their achieved outcomes. Information was sought to determine whether there are differences of productivity across the six regions, in an effort to understand what improvements might be needed, and whether improvements should be initiated at the national or region-by-region basis.

Key findings are that herd size is generally small (Table 1); with an average of 4 dairy animals per farm, of which 2.6 were actually in milk. The Tanga region had somewhat larger herds, at 6.8 dairy animals per farm. Milk yields per cow were modest (Table 2), at an average of 12.6 L/cow/day for those animals that were currently lactating. Only data for current daily yield were available, but data on annualised yields were not obtainable. Nonetheless, there was little variation in yields per cow across regions, suggesting that the underlying constraints to productivity were likely to be similar across regions. Prominent amongst these constraints were low bodyweight (average of 346 kg; similar across regions) and moderate BCS (median of 3.0; again, similar across regions). The proportion of cows that were pregnant at the time of the “snapshot” pregnancy diagnosis (Table 6) was low, at only 46% of all dairy cattle; and, although there were small numerical differences in the pregnancy rate across regions, these were not statistically significant. The proportion of nulliparous heifers that were pregnant (55%), was substantially higher than that of parous animals (43%: Table 6). Although a higher pregnancy rate in nulliparous than parous cows is widely reported in the literature (e.g. Ukita et al. 2022), pregnancy rates for nulliparous animals are commonly > 80%, meaning that the present results were low by global standards (de Oliveira Marques et al. 2015).

Most of the present results, with the exception of pregnancy status, were based on farmer records and/or recollections. There was a wide variability in the level and standard of record keeping, with most documentation being informal, characteristically just on paper or displayed on walls. This clearly affected the quality and availability of reproductive records, with consequent adverse effects upon the accuracy, consistency and reliability of the data. Although most farmers attempted to track basic herd information, key events such as the dates of breeding and calving were often left unrecorded, particularly if male calves were born, calves died early, or breeding occurred multiple times. In such cases, farmers relied more on memory or verbal accounts rather than records, increasing the risk of inaccuracies. Similar issues were reported by Kanuya et al. (1997), so it seems that the situation has not substantially improved in the last 25 years. The accuracy of the data was a particular issue relating to conceptions to first service (Table 5), inasmuch as simply accepting these data as correct would have indicated a 73% conception rate to first service, a rate which is inimical with international literature (Lucy 2001; Diskin and Morris 2008) on conception rates in parous dairy cows. The most likely reason for this high apparent conception rate is that farmers only remembered the last service date, thus rendering FSCI of no use as a parameter of assessment of reproductive performance. CFSI might be of more use, although similar constrains to its value to those that limit FSCI would presumably pertain. However, the issue is not just one of accuracy, inasmuch as only < 50% of the cows that were presented for pregnancy testing had sufficient records available for evaluating the four other key reproductive parameters (i.e., CFSI, FSCI, CCI and ICI) (see Table 4).

The definitive outcome parameter, ICI, is the key indicator of a herd’s reproductive performance (Fetrow et al. 2007), and as such, provides a simple comparison across studies, regions and countries. The present study reported an ICI with mean and median values of 563 and 468 days, respectively (range: 398 to 686 days) (Table 4). Notably, cows in the Kilimanjaro and Morogoro regions had a median ICI exceeding 550 days, which was ~ 1.5x the target of 365 days that equates to producing one calf per year. These findings are consistent with previous smaller-scale studies of smallholder dairy farms in Tanzania, which have shown similarly prolonged ICI: 477 days (Kanuya et al. 2000); 500 days (Swai et al. 2005b); 480 days (Asimwe and Kifaro 2007); 476days (Swai et al. 2007) and in African countries, an ICI of up to 33 months (Allan et al. 2024). However, as Fetrow et al. (2007) state, the large timespan needed to get two calving dates means that it is of limited value as a monitoring tool. This is particularly a problem in systems such as Tanzanian smallholder dairy farms where the ICI is prolonged, and because its calculation relies on two sets of farmer-recorded data (i.e. two successive calving dates). In the present survey, only 33% of cows had two calving dates recorded by the farmer. Furthermore, ICI relies completely on farmer records, so if two calving dates have not been recorded for a multiparous cow, ICI data will be unobtainable. Unsurprisingly, therefore, relying on farmers’ recollections for dates of events that occurred over 350 days ago (and in many cases, over 500 days ago) is likely to be inaccurate.

The ICI is itself composed of the shorter-term intervals VWP, CFSI, FSCI and CCI, plus the length of gestation. Monitoring these may be more effective than ICI, as they do not require data for two successive calvings. The CCI is therefore often recommended as a key method for determining a farm’s reproductive efficiency because of its simplicity and speed relative to ICI (Hudson et al. 2018). In order to achieve a 365-day inter-calving interval, CCI should average 85 days (Esslemont 1992; Plaizier et al. 1997, 1998), but in the present study, the length and variability of the CCI (mean and median 268 and 189 days, respectively; range 38-1458 days) exceeded this and so is clearly likely to underpin the length and variability of the ICI. Unfortunately, however, CCI data were retrievable from only a low proportion (24%) of animals, as calculating a CCI requires that a cow is pregnant, has a previous (recorded) calving date, and that the date of the breeding to which the cow conceived is recorded. In the present study, only 65% of the pregnancy-tested cattle had a recorded previous calving date, and of these, only 38% were pregnant. Hence, CCI data, whilst valuable, was difficult to obtain for enough animals to provide useful information at the farm level.

Despite these uncertainties, it could be considered that, in cows that have been pregnancy-tested and have an estimate of gestational age, CCI could be relatively useful, inasmuch as it only requires a calving date (which farmers are likely to remember relatively accurately even when it is not recorded) and an estimate of conception date. If so, the median CCI and interquartile range (IQR: 111–334 days) reported in the present study may reflect the true distribution of CCI in smallholder dairy cattle in Tanzania, especially as the present data are consistent with earlier studies in Tanzania, which also reported prolonged CCI (i.e. 122 days: Kanuya et al. 1997, p. 123 days: Lyimo et al. 2004; and 221 days: Kanuya et al. 2000). On the other hand, it is likely that the maximum reported CCI (1531 days) was an underestimate, given that the maximum reported ‘CFSI’ was 1822 days. However, the regional median CCIs probably reflect the true distribution of CCI across all cows (i.e. including those for which no records were obtainable) in smallholder dairy cattle in those regions. Modelling of CCI using survival analysis did not identify clear differences in CCI between most regions. Hence, this analysis highlights the importance of the farm, even within the region, as the main factor determining reproductive outcomes and the need for more data to properly evaluate the impact of the region on CCI.

The major components of the CCI are, in turn, the VWP, CFSI, oestrus detection rate and conception rate (Hudson et al. 2012). Conception rate and oestrus detection rate could not be directly assessed either from records or by on-farm observation, but farmers were able to state the VWP for their herds. To obtain an 85-day CCI (i.e. a 365-day ICI), a VWP of 45 to 60 days is generally recommended, with 90% of cows having a VWP of < 10 weeks (Inchaisri et al. 2011). In the present study, the mean and median VWP over the entire study cohort were 99.6 and 90 days respectively (range: 35–360 days); and only in Mbeya was the median stated VWP < 90 days (i.e. 60 days: Table 3). Further, over all regions, only 62/320 farmers stated that their VWP was ≤ 70 days. Moreover, there was concern over the accuracy of farmers’ reporting of their VWP, as 52/313 cows with calculable CCIs had intervals that were shorter than the VWP reported for their farm. Regression analysis of VWP data against CCI data showed that an increase in VWP from 50 to 125 days resulted in an increase in CCI of 30 days. Remarkably, this differs from the effect of extending VWP in intensively managed dairy herds, in which the same extension of the VWP results in an increase of approximately 76 days open (Ma et al. 2022). It is unclear how much attention farmers involved in the present study had paid to their reported VWP, or whether the concept of VWP and its application to their farms is clear to Tanzanian smallholder farmers. The use of the alternative measure of “days open” (Fetrow et al. 1990) might be considered to circumvent this problem, but similar difficulties would pertain, as, although days open includes data from non-pregnant cows, its calculation still requires that the interval from calving to culling or death is known. Those data generally did not exist.

The final major component of CCI that was assessed in the present study was CFSI. The CFSI varied from 28 to 1822 days, with mean and median values of 249 and 166 days, respectively (Table 4), with little regional variation. The long CFSI interval undoubtedly reflects the long and variable VWP, but is likely to also reflect poor oestrus detection and poor conception rates. Poor oestrus detection is a well-known problem of small herds (Diskin and Sreenan 2000; Ojango et al. 2017; Allan et al. 2024; Ngou 2026), so may be as much a characteristic of herd size as it is of management deficiencies. Low conception rate is also a common problem of European cattle in (sub)tropical environments (Syrstad and Ruane 1998; Hemme and Otte 2010) and may also reflect the intrinsic limitations of using Holstein-derived animals (Ngou et al. 2025) in such an environment, rather than necessarily indicating management deficiencies. On the other hand, Ngou et al. (2025) also identified a problem of inadequate oestrus detection amongst Tanzanian smallholder farmers, as well as seasonal feed shortages and poor efficiency of the AI service.

By contrast to the foregoing measures that required farmers to accurately record breeding data, pregnancy diagnosis by transrectal ultrasound was undertaken for every cow in the survey, and, by re-examining animals that appeared to be non-pregnant at the first examination 28 days later, an accurate data set for the proportion of pregnant animals could be generated. Furthermore, measuring pregnancy status using transrectal ultrasound does not need farm records. It simply requires a skilled technician to go onto the farms and for the farmers to present all eligible cows for examination. It is particularly useful for cows that are brought in with an unknown history, which is not an uncommon situation among smallholder dairy farmers in Tanzania. Further, the proportion of pregnant cattle on the farm reflects reproductive success; if more cattle become pregnant sooner and fewer fail to become pregnant, then the proportion of pregnant cattle will increase. As such, repeated strategic measurements of the proportion pregnant could provide an ongoing monitoring of reproductive performance, with targets set based on a baseline that are perhaps derived from those reported in the present study. An additional advantage of monitoring reproduction using pregnancy diagnosis is that it is very popular with farmers. Many farmers said that they were very pleased to learn the pregnancy status of their animals, especially in cows, as they often had no records. The absence of records was not only due to frequent purchases from undocumented sources but also originated from limited awareness of the importance of proper farm record keeping, reliance on memory and the lack of standardised systems for tracking reproductive events. Pregnancy testing thus provided farmers with information that they could directly use in the future. Some farmers even accompanied the first author to nearby farms to help others access the pregnancy testing service and to learn from successful peers, providing support to those facing challenges. The other advantage of pregnancy diagnosis is that its value is increased if farms have records (even if they are limited in nature). Promoting pregnancy diagnosis can thus be used as a method of promoting record keeping.

Comparison of the present results for the proportion of pregnant animals with the results in the literature is difficult, inasmuch as there are very few equivalent studies that report this parameter. Most East African studies that have reported proportions of pregnant cattle have actively measured pregnancy rates in a specified cohort (e.g. inseminated cows: Nishimwe et al. 2015, percentage pregnant after first service: Kanuya et al. 2000; lactating cows: Kanuya et al. 2006; or synchronised cattle: Kabuni et al. 2025). Studies that have measured the proportion of cows pregnant in an unselected group of cows include: Muraya et al. (2018) and Mungube et al. (2019) in Kenya (29 and 46% of cows pregnant, respectively) and Lobago et al. (2006) in Ethiopia (41%). Nonetheless, it would appear that the present finding that 46% of the cattle presented for examination were pregnant is at the upper end of previously reported proportions and, further, the overall pregnancy rate varied by region (see Table 6) and between cows and heifers. The effect of region was particularly marked for heifers, which were more likely to be pregnant than cows. Further, these regional differences could reflect varying access to breeding services and feed availability. More data are needed on what is driving the difference between heifers and cows and between regions, but it is likely that a high proportion of pregnant heifers, especially in Mbeya, reflects the widespread use of natural service.

## Conclusion

The overall reproductive performance of smallholder dairy cattle across the six primary dairy-farming regions of Tanzania was poor. Overall pregnancy rate was low (46%), ICI was long (mean/median: 563/468 days) and CCI was long (mean/median: 268/189 days). Pregnancy rate was somewhat higher in nulliparous than parous cows (55% vs. 43%), but neither were adequate. Record-keeping practices were generally unsatisfactory, with most farms relying on farmer recall. Key reproductive parameters could be calculated for only 24–46% of animals. Conversely, direct physical pregnancy diagnosis produced data for 100% of animals (parous and nulliparous) in the study cohort, and was regarded by farmers as an invaluable source of data. These findings highlight the urgent need for improved record-keeping systems and reproductive management at farm level. The use of the “proportion pregnant” (i.e. after physical pregnancy diagnosis) is recommended as an interim standard reproductive performance indicator until record-keeping improves. This should be implemented alongside with targeted training on simple farm record keeping practices, as well as region tailored interventions on dairy farm management.

## Data Availability

The generated and analysed datasets used in this study are available from the corresponding author upon acceptable request.

## Abbreviations

TALIRI: Tanzania Livestock Research Institute
DC: District Council
TC: Town Council
MC: Municipal Council
CC: City Council
IMB: International Business Machines
CI: Confidence Interval
OR: Odds Ratio
HR: Hazard Ratio

## Declarations

### Footnotes

Ngou et al.
